# Relative validation of the dietary intake of fatty acids among adults in the Swedish National Dietary Survey using plasma phospholipid fatty acid composition

**DOI:** 10.1017/jns.2015.1

**Published:** 2015-06-26

**Authors:** Eva Warensjö Lemming, Cecilia Nälsén, Wulf Becker, Peter Ridefelt, Iréne Mattisson, Anna Karin Lindroos

**Affiliations:** 1 National Food Agency, Uppsala, Sweden; 2 Department of Medical Sciences, Clinical Chemistry, Uppsala University, Uppsala, Sweden

**Keywords:** Fatty acid intake, National dietary studies, Swedish National Dietary Survey, Phospholipids, Plasma biomarkers, LA, linoleic acid, NFA, National Food Agency, PL, phospholipid

## Abstract

The aims of a national dietary study are several-fold. One purpose is to monitor the intake of foods and nutrients in the population and to compare intakes with dietary recommendations. It is, however, difficult to measure dietary fat intake and plasma biomarker fatty acid (FA) composition may be used as an objective measure of dietary fat intake. Thus, the relative ability of a diet record to capture habitual fat intake was validated against biomarker FA. Dietary fat intake was measured in a novel self-assisted web-based 4-d food record – the ‘Riksmaten’ method. Spearman rank correlations between dietary FA, certain food groups (fish-shellfish, dairy products, meat and sausages, and spreads) and the fat content of these food groups and biomarker FA were explored. Participants were 150 women and 129 men, aged 18–80 years, who took part in the Swedish National Dietary Survey, Riksmaten adults 2010–11. Blood samples were collected on average 20 d after the diet record and FA composition was measured in plasma phospholipids by GLC. Total *n*-3 FA (*r* 0·31), EPA (*r* 0·34) and DHA (*r* 0·42) were correlated between plasma and diet (all *P* ≤ 0·001). Adjustment for covariates attenuated the relationships. Linoleic acid was only marginally correlated (*r* 0·15; *P* = 0·06) in women. Plasma pentadecaenoic acid and heptadecaenoic acid were correlated with dairy product intake as previously reported. In conclusion, the Riksmaten method appears valid for the purpose of collecting data on dietary fat composition, at least in a healthy adult population.

There is a continuous interest in dietary fats and their association with chronic disease and especially CVD^(^^1^^)^. Dietary fatty acids are biologically active compounds that participate in various pathways and regulate the fluidity of biological membranes. Further, PUFA regulate gene expression and are precursors of eicosanoids^(^^2^^)^. The current dietary recommendations advise us to decrease the intake SFA and simultaneously to increase the intake of unsaturated fatty acids^(^^3^^)^ to reduce the risk of CVD^(^^4^^)^ and is a cornerstone in public health nutrition. One main dietary advice in Sweden^(^^5^^)^ is to consume fish 2–3 times per week (of which one portion should be fatty fish) since fish consumption is associated with established cardioprotective benefits^(^^6^^)^ and contributes essential nutrients, such as *n*-3 fatty acids (EPA and DHA) and vitamin D. It is recommended that *n*-3 fatty acids account for a minimum of 1 % of energy intake^(^^3^^)^.

In order to monitor and evaluate the intake of, for example, fish and *n*-3 fatty acids in the population the results from the Swedish National Dietary Survey is used. In the latest survey, Riksmaten adults 2010–11^(^^7^^)^, a newly developed web-based self-assisted 4 d dietary record was used to collect diet data. A diet record or a 24 h recall is considered to give more accurate dietary intake data than, for example, FFQ^(^^8^^,^^9^^)^. However, in any dietary survey, including our national dietary survey, there is an inherent difficulty in measuring dietary exposure and especially dietary fat^(^^10^^,^^11^^)^. This is connected to random and systematic measurement errors, the difficulty in estimating portion sizes as well as inadequacies in food composition databases, for example the use of standard fat in recipes. Under-reporting is common in all dietary survey techniques and may lead to spurious diet–disease relationships^(^^12^^)^. Moreover, the degree of under-reporting is affected by different factors, for example the BMI among participants, and this is particularly true for foods perceived as unhealthy^(^^10^^–^^13^^)^.

Biomarker fatty acids may be used as indicators of dietary fat. However, fatty acid biomarkers do not provide an accurate reflection of absolute intake, partly because fatty acid composition is influenced by endogenous metabolism and remodelling^(^^14^^,^^15^^)^ as well as to sex, genotype, fat intake level, BMI, smoking status and exercise^(^^16^^)^. Certain fatty acids such as linoleic acid (LA; 18 : 2*n*-6), EPA and DHA provide a better reflection of the dietary content, while others such as SFA and MUFA provide a worse estimate of dietary intake because of endogenous metabolism. The biomarker fatty acid composition in the phospholipid (PL) and cholesteryl ester fraction of lipoprotein particles reflects the dietary fat quality during the last days and weeks, respectively, while fatty acids of other fractions such as erythrocyte membranes and adipose tissue reflect the intake of those fatty acids during the last months and year(s), respectively^(^^15^^,^^17^^–^^21^^)^. Thus, the relative validity of the intake of dietary fatty acids from a dietary survey may be validated using biomarker fatty acids.

The novel web-based Riksmaten method, a 4-d web-based dietary record used in the latest Swedish National Dietary Survey, Riksmaten adults 2010–11, is currently being validated. The present study aimed to investigate the relative validity of the estimated dietary intake of fat, especially EPA and DHA and LA, using biomarker fatty acids measured in plasma PL in a subsample of Riksmaten adults 2010–11. We also investigated associations between intake of certain food groups and amount of fat from the food groups – fish and shellfish, spreads (margarines, dairy-based spreads and butter), meat and sausages and dairy products (milk, yoghurt, cream, sour cream and cheese) – and corresponding biomarker fatty acids in plasma PL. In addition, the ability of the questionnaire to capture intake of *n*-3 fatty acids from fish-shellfish intake was explored.

## Experimental methods

### Study design and population

**Fig. 1.**
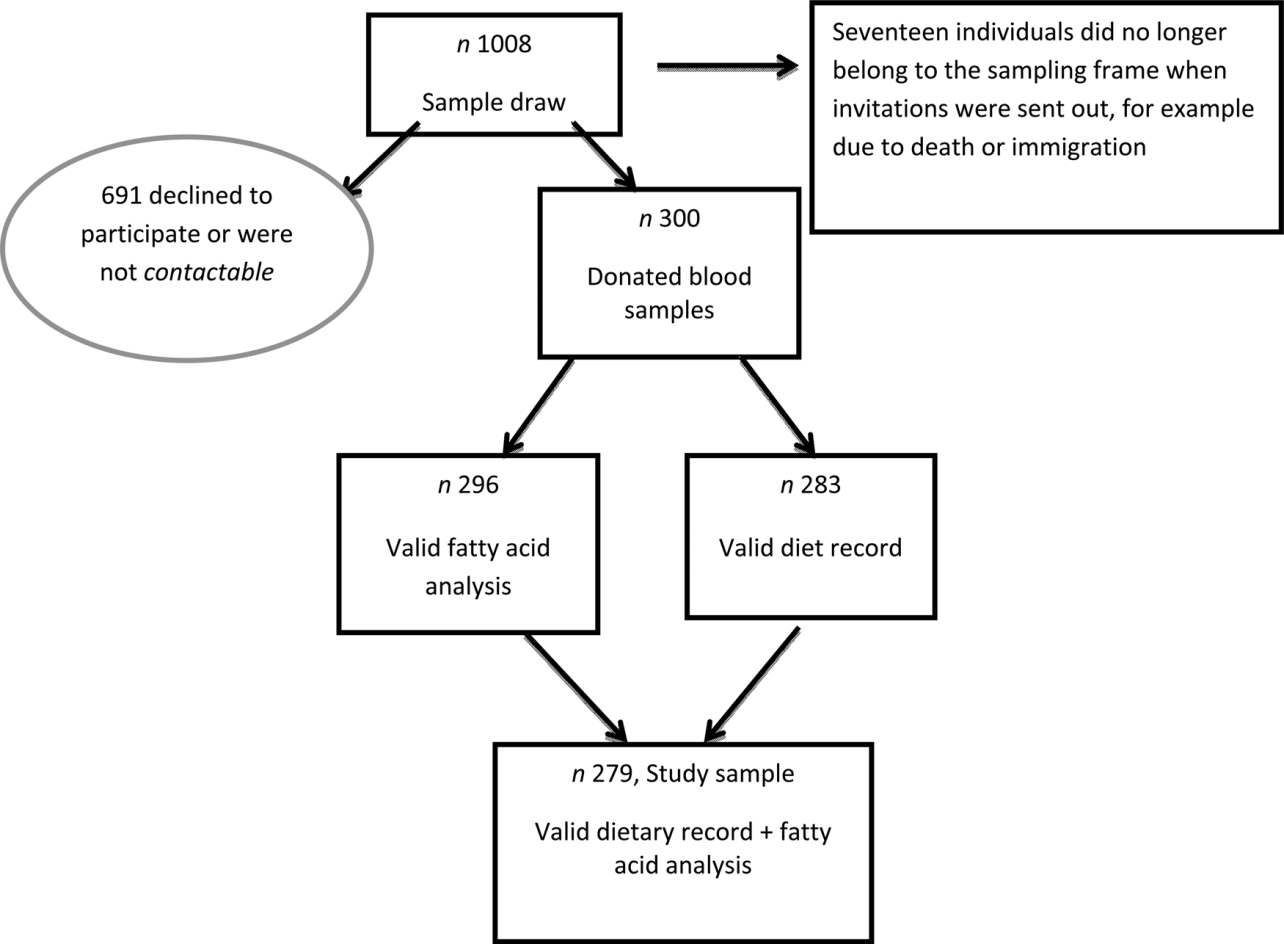
Flowchart depicting the study sample. The study sample of 1008 refers to the subsample in the Riksmaten adults 2010–11 study.

Riksmaten adults 2010–11 is the latest Swedish National Dietary Survey conducted by the National Food Agency (NFA) in association with Statistics Sweden. The study was carried out between May 2010 and July 2011 to capture seasonal variations. A nationwide random sample (*n* 5003) was selected and 36 % (1005 women and 792 men) partook in the dietary survey^(^^7^^)^. The invited sample was also requested to complete a questionnaire. In a subsample (*n* 1008) blood and spot urine samples were collected to measure markers of nutritional status and exposure to toxic contaminants (see Fig. 1). All participants in the present study completed the diet record, the questionnaire and donated blood samples on average 20 d after the dietary registration was initiated.

Sweden has seven Occupational and Environmental Medicine Centre regions. From each of these seven regions, the regional capital (Linköping, Lund, Stockholm, Umeå, Uppsala, Gothenburg, Örebro) and two randomly chosen municipalities were chosen and an equal number of individuals was randomly selected in each region independent of population size. Altogether, 1008 individuals aged between 18 and 80 years old were invited to participate in the blood sampling (twelve individuals per municipality) evenly distributed over the year. The selection of study participants was performed by Statistics Sweden, and the Occupational and Environmental Medicine Centres carried out the recruitment of the participants. In total, 300 individuals provided blood samples (participation rate 30 %; see Fig. 1). The study was approved by the local ethics committee at Uppsala University and all participants gave oral consent to participate.

### Dietary assessment

The diet record ran over four consecutive days and was entered by the participants in a web-application (self-assisted), developed by the NFA. A public version of this is available at http://www.slv.se/sv/grupp1/Mat-och-naring/kostrad/Test-matvanekollen/ (in Swedish). The starting day was randomly allocated for the participants to capture consumption on all days of the week. Participants were instructed to record everything that they ate and drank during these days. The food list in the web application contained more than 1900 food items and dishes. A portion guide, household measures, numbers of portions (cups, pieces, slices) and grams were used to estimate the amounts eaten. The portion guide included twenty-four different food categories, with four to eight different reference sizes in each category. The application (version 04.1) is linked to the national food composition database (Livsmedelsdatabasen, version Riksmaten adults 2010–11) at the NFA, which enables automatic estimation of nutrient values and energy intake. Intake of food groups (g), energy and nutrient intakes per d were calculated as the average intake over the four record days. Information on physical activity level at work and leisure time was collected as part of the dietary record on a four-grade scale. Under-reporting was calculated using the Goldberg cut-off according to Black^(^^22^^)^ in the entire Riksmaten survey population and defines the probability of adequately reported energy, based on body size and physical activity level.

### Questionnaire and other data

The questionnaire was used to collect background data, other characteristics and information on foods less frequently consumed (episodic foods). Information on self-reported weight and height was also collected in the questionnaire. BMI was calculated as weight (in kg) divided by height (in m) squared. The consumption frequency of different types of fish and shellfish during the previous year was collected. The information from this question was used to create a three-level categorical weekly fish intake variable, with the levels <1 time per week, 1–2 times per week, >2 times per week. The consumption frequency (ranging from never to every day)^(^^23^^)^ of salmon, lean white fish (for example cod and saithe), herring and mackerel, and shrimp, crayfish and lobster was used to create this variable. Information on supplement use of fish oil and *n*-3 capsules reported in the questionnaire was used in sensitivity analysis. Data on age and sex of participants were collected from the Swedish population register.

### Sampling of blood

Non-fasting blood was sampled at the Occupational and Environmental Medicine Centre in each region. Blood was drawn from an antecubital vein. Plasma was separated and frozen (–20°C) at the regional centres. The samples were then transported frozen to the Department of Clinical Chemistry, University Hospital Uppsala and then distributed via the NFA for analyses.

### Analysis of plasma fatty acid composition in phospholipids

Fatty acid composition in plasma PL was analysed at Uppsala University as previously described^(^^24^^)^. Briefly, methanol was added to plasma for lipid extraction. PL were separated by TLC before inter-esterification with acidic methanol at 85°C for 2 h. To avoid contamination of the GLC column, non-esterified cholesterol liberated in the reaction was removed by an aluminium oxide column. The percentage composition of methylated fatty acids (14 : 0 to 22 : 6) was determined by GC with a flame ionisation detector and He as the carrier gas. A previous larger population study concluded that fasting state did not influence associations between fatty acids in PL and diet^(^^25^^)^.

### Statistical analysis

The normality of the variables was checked with the Shapiro–Wilk test. Non-normal variables were log-transformed to improve normality. Plasma fatty acids in PL are expressed as weight percentage of total fatty acids. Food group and nutrient intakes were adjusted for total energy intake using the residual method^(^^23^^)^. The investigated food groups were fish and shellfish, spreads, meat and sausages, and dairy products (milk, yoghurt, fermented milk cream, sour cream and cheese). The spreads were margarines, dairy-based spread and butter used on bread or as a side dish (for example on boiled potatoes). The intake of fish-shellfish and dairy products, combined with data on fat content, was used to derive the amount of fat (in g/d) from the respective food group. The dietary intake of individual fatty acids (weight percentage) were treated as the percentage of total fat intake since this is conceptually similar to the way that fatty acids in plasma PL are expressed^(^^25^^,^^26^^)^.

The crude relationship between plasma fatty acids and dietary fatty acids, food groups and fat from food groups as well as fish-shellfish intake from the questionnaire was tested with Spearman rank correlations. Partial correlation coefficients were adjusted for BMI (continuous variable), smoking habits (categorical variable), physical activity level (categorical variable), alcohol intake and age (both continuous variables). In order to test for effect modification by sex we analysed the association between fatty acids in plasma and diet with linear regression analysis and introduced an interaction term between dietary fatty acid and sex.

Differences in characteristics between women and men were tested by *t* test, χ^2^ test and the Wilcoxon rank-sum test. ANOVA was used to test for group differences and analyses were stratified on sex. The agreement between the diet record and questionnaire to rank individuals in terms of fish-shellfish intake (tertiles) was evaluated in weighted kappa (κ_w_) statistics.

The influences of under-reporting and supplement use (fish-oil and *n*-3 capsules) were accounted for in sensitivity analyses. *P* values <0·05 was considered significant. All analyses were carried out in STATA version 12.1 (STATA Corp.).

## Results

### Background and dietary characteristics

Table 1 depicts characteristics of study participants. More women (*n* 150; 54 %) than men (*n* 129; 46 %) participated in the study and women were on average younger. Physical activity level, smoking habits and number of under-reporters did not differ between the sexes. There were small differences in food intake and fat intake (expressed as g/10 MJ) between men and women, except that women had a higher intake of dairy products (*P* = 0·03) and men had a higher intake of spreads (*P* < 0·01). In relation to the other participants in the Swedish National Dietary Survey (*n* 1518) the intakes of protein, carbohydrate, dietary fibre, alcohol and total fat (energy percentage) were not different (data not shown). However, the participants who donated blood samples reported significantly higher energy intake (8·6 *v.* 8·2 MJ; *P* = 0·004). The relative proportions of fatty acids measured in plasma PL are depicted in Table 2. Age was positively associated with the proportions of plasma PL EPA and DHA while negatively associated with plasma LA, α-linolenic acid and oleic acid. About 20 % of the participants had not consumed fish according to the diet record.

**Table 1.** Characteristics of study participants (Number of subjects and percentage, mean values and standard deviations, and medians and interqurtile ranges (IQR))*

	All		Women		Men		
	Median	IQR	Median	IQR	Median	IQR	*P* for difference
Subjects							
*n*	279		150		129		
%			54		46		
Age							0·03
Mean	50		48		53		
sd	17		16		17		
BMI (kg/m^2^)	24·8	22·6–27·5	24·2	22·3–27·5	25·1	23·3–27·5	0·06
Physical activity level							
At work							0·31
Sedentary							
*n*	209		116		93		
%	75		75		72		
Heavy							
*n*	70		34		36		
%	25		25		28		
At leisure time							0·99
Sedentary							
*n*	132		71		61		
%	47		47		47		
Active/regular							
*n*	147		79		68		
%	53		53		53		
Current, occasional smokers (%)	15		17		13		0·45
Supplement use (fish oil, *n*-3)							0·17
*n*	46		29		17		
%	16		19		13		
Under-reporters (%)	13·6		14·0		13·2		0·84
Energy (MJ)							<0·001
Mean	8·6		7·4		9·4		
sd	2·4		2·0		2·5		
Dietary fat intake							
Total fat (g/10 MJ)	91	82–104	92	82–104	90	82–103	0·66
SFA (g/10 MJ)	35	30–40	35	30–41	35	30–39	0·73
% Total fatty acids							0·80
Mean	38		38		38		
sd	5		5		6		
MUFA (g/10 MJ)	34	30–39	34	30–38	34	30–39	0·91
% Total fatty acids							0·44
Mean	37		37		37		
sd	3		3		3		
PUFA (g/10 MJ)	14	12–18	14	12–18	14	12–17	0·68
% Total fatty acids							0·98
Mean	16		16		16		
sd	5		4		5		
Fat from dairy products (g/10 MJ)	6·7	3·2–10·3	6·9	3·7–11·3	6·0	2·7–9·5	0·17
Fat from fish and shellfish (g/10 MJ)	4·3	0·3–9·5	4·3	0·4–8·7	4·3	0·1–10·3	0·75
Intake of food groups							
Fish and shellfish (g/10 MJ)	44	4·5–77	43	5·6–74	47	4·5–78	0·82
Dairy products† (g/10 MJ)	319	183–431	346	216–453	279	167–422	0·03
Meat and sausages (g/10 MJ)	99	67–138	97	60–131	104	72–154	0·07
Spreads‡ (g/10 MJ)	11	3·9–20	9	1·3–17	14	5·5–23	<0·01

*Mean and standard deviation are reported for normally distributed variables and as median and interquartile range (25th and 75th percentile] for skewed variables.

†Dairy products include milk, yoghurt, fermented milk, sour cream, cream and cheese.

‡Spreads include different margarines, spread mixtures and butter used on bread or as a side dish (for example on boiled potatoes).

**Table 2.** Proportion (% of total) of fatty acids estimated in plasma phospholipids in all participants (*n* 279) and in women (*n* 150) and men (*n* 129) (Mean values and standard deviations)

	All		Women		Men	
Fatty acid	Mean	sd	Mean	sd	Mean	sd
∑ SFA	47·2	1·6	47·1	1·6	47·3	1·7
Myristic acid, 14 : 0	0·41	0·1	0·43	0·10	0·40**	0·09
Pentadecanoic acid, 15 : 0	0·26	0·05	0·27	0·05	0·26	0·06
Palmitic acid, 16 : 0	31·3	1·5	31·2	1·5	31·4	1·5
Heptadecanoic acid, 17 : 0	0·43	0·06	0·43	0·06	0·42	0·07
Stearic acid, 18 : 0	14·8	1·2	14·8	1·2	14·8	1·2
∑ MUFA	13·5	1·2	13·5	1·3	13·5	1·2
Palmitoleic acid, 16 : 1*n*-7	0·50	0·2	0·53	0·16	0·49***	0·21
Oleic acid, 18 : 1	13·0	1·1	12·9	1·2	13·2	1·4
∑ PUFA	39·3	2·0	39·4	1·9	39·2	2·1
∑ *n*-6	32·5	2·2	32·6	2·1	32·3	2·2
Linoleic acid, 18 : 2*n*-6	21·3	2·3	21·6	2·4	20·9	2·4
γ-Linolenic acid, 18 : 3*n*-6	0·11	0·04	0·10	0·04	0·1	0·04
Dihomo-γ-linolenic acid, 20 : 3*n*-6	2·8	0·6	2·8	0·7	2·8	0·6
Arachidonic acid, 20 : 4*n*-6	8·3	1·4	8·2	1·5	8·5	1·4
∑ *n*-3	6·8	1·7	6·7	1·8	6·9	1·6
α-Linolenic acid, 18 : 3*n*-3	0·28	0·1	0·29	0·1	0·27*	0·08
EPA, 20 : 5*n*-3	1·7	0·8	1·7	0·9	1·7	0·7
Docosapentaenoic acid, 22 : 5*n*-3	0·93	0·2	0·91	0·18	0·96*	0·17
DHA, 22 : 6*n*-3	3·9	1·0	3·9	1·1	3·9	0·98

Mean value was significantly different from that for men: *P ≤ 0·05, **P ≤ 0·01, ***P ≤ 0·001.

### Correlations between plasma and dietary fatty acids

The crude Spearman rank correlations between proportions of fatty acids in plasma PL and dietary fatty acids are presented in Table 3. In general, plasma *n*-3 fatty acids showed the strongest crude correlations with diet: *r* 0·34 for EPA and *r* 0·42 for DHA (all *P* ≤ 0·001). Myristic acid (14 : 0) (*r* 0·16; *P* < 0·01) as well as total PUFA (*r* 0·12; *P* = 0·05) were correlated between PL and diet. PL LA (18 : 2*n*-6) was not significantly correlated with dietary intake (*r* 0·07; *P* = 0·24). Repeating these analyses with the dietary fatty acids expressed as g/d or residual adjusted g/d rendered similar results (data not shown). The relationships for myristic acid (14 : 0), total MUFA and PUFA and LA were only significant in one of the sexes. Further, for palmitoleic acid, total PUFA and LA the correlation coefficients went in opposite directions in men and women and this was confirmed as significant interactions with sex in the linear regression analysis: palmitoleic acid (*P* for interaction = 0·028), total PUFA (*P* for interaction = 0·031) and LA (*P* = 0·034).

**Table 3.** Spearman rank correlations (*r*) between fatty acid intake estimated in the diet by a 4-d food record and plasma phospholipids, in all study participants (*n* 279) and by sex†

Fatty acid	All	Women	Men
Total SFA	–0·07	–0·08	–0·07
Myristic acid, 14 : 0	0·16**	0·10	0·21*
Palmitic acid, 16 : 0	–0·02	–0·07	0·05
Stearic acid, 18 : 0	–0·03	0·04	–0·13
Total MUFA	–0·11	–0·04	–0·21*
Palmitoleic acid, 16 : 1	–0·03	0·10	–0·10
Oleic acid, 18 : 1*n*-7	–0·10	–0·04	–0·16
Total PUFA	0·12*	0·17*	0·07
Total *n*-6 fatty acids	0·10	0·08	0·13
Linoleic acid, 18 : 2*n*-6	0·07	0·15	–0·03
Arachidonic acid, 20 : 4*n*-6	0·07	0·10	0·02
Total *n*-3 fatty acids	0·31***	0·33***	0·28***
α-Linolenic acid, 18 : 3*n*-3	0·07	0·09	0·05
EPA, 20 : 5*n*-3	0·34***	0·36***	0·28***
Docosapentaenoic acid, 22 : 5*n*-3	0·05	0·06	0·009
DHA, 22 : 6*n*-3	0·42***	0·44***	0·39***

*P ≤ 0·05, **P ≤ 0·01, ***P ≤ 0·001.

†Pentadecanoic acid (15 : 0), heptadecanoic acid (17 : 0), γ-linolenic acid (18 : 3*n*-6) and dihomo-γ-linolenic acid (20 : 3*n*-6) could not be determined in the diet and were thus not correlated in this analysis.

Adjusted correlations (partial) between plasma PL and dietary fatty acids were in most cases attenuated. Total *n*-3 fatty acids (*r* 0·19; *P* < 0·01) as well as EPA (*r* 0·18; *P* < 0·01) and DHA (*r* 0·33; *P* < 0·001) remained correlated. The negative correlation between total MUFA in plasma and diet was stronger after adjustment (*r* –0·17; *P* = 0·006) and for individual SFA most correlations remained unchanged. In men, the total SFA amount in plasma was inversely related to diet (*r* –0·18; *P* = 0·01) after adjustment.

### Correlations between plasma fatty acids and food groups

#### Dairy products

The intake of dairy products (g/d) correlated with plasma PL 14 : 0 (*r* 0·15; *P* < 0·01) and 15 : 0 (*r* 0·25; *P* < 0·001) but not with 17 : 0 (*r* 0·08; *P* = 0·2) in all participants, and differed between men and women. Adding butter (recorded as a spread by twenty-three participants) to the dairy product variable did not change the correlations (data not shown). Total fat and SFA intakes from dairy products were significantly correlated with plasma PL 15 : 0 and 17 : 0, but not with 14 : 0 and correlations differed between the sexes (Table 4). The partial correlation analyses showed that PL 15 : 0 remained and 17 : 0 became significantly correlated with the intake of dairy products in women. Further, the correlations between intake of total fat and SFA from dairy products disappeared for 15 : 0 but remained for 17 : 0 in women. In men, plasma PL 15 : 0 was correlated with the intake of dairy products as well as with the intake of total fat and SFA from dairy products (Table 4). PL 15 : 0 and 17 : 0 did not correlate with dietary fish-shellfish intake (data not shown).

**Table 4.** Crude and adjusted† Spearman rank correlations (*r*) between specific plasma phospholipid fatty acids and dairy products in all study participants (*n* 279) and by sex

		Dairy products‡ (g/d)			Total fat from dairy products‡ (g/d)			SFA from dairy products‡ (g/d)		
Fatty acid		All	Women	Men	All	Women	Men	All	Women	Men
14 : 0	Crude	0·15**	0·08	0·19*	0·07	–0·006	0·12	0·008	–0·007	0·13
	Adjusted	0·12	0·10	0·15	0·02	0·03	0·03	0·02	0·03	0·04
15 : 0	Crude	0·25***	0·10	0·36***	0·32***	0·20**	0·43***	0·31***	0·18*	0·44***
	Adjusted	0·22***	0·24*	0·19*	0·09	0·07	0·16	0·10	0·06	0·18*
17 : 0	Crude	0·08	0·10	0·05	0·16**	0·17*	0·15	0·16**	0·16*	0·15
	Adjusted	0·11	0·21**	–0·06	0·10	0·20*	–0·04	0·11	0·19*	–0·02

*P ≤ 0·05, **P ≤ 0·01, ***P ≤ 0·001.

†Adjusted for BMI, smoking habits, physical activity level, alcohol intake and age.

‡Residual adjusted.

#### Fish and shellfish

Intakes of total fish-shellfish and total fat and PUFA from fish- shellfish from the diet record were well correlated with very-long-chained *n*-3 fatty acids in PL. The strongest correlation was between DHA and total fat intake from fish-shellfish (*r* 0·38; *P* < 0·001). The intake of fish-shellfish reflected as the weekly fish intake correlated well (all *r* ≥ 0·41 and *P* < 0·001) with total *n*-3 PUFA, EPA and DHA in plasma PL (Table 5). Correlations between dietary fish-shellfish intake and total *n*-3 (*r* 0·24; *P* < 0·001), EPA (*r* 0·19; *P* < 0·01) and DHA (*r* 0·29; *P* < 0·001) in PL were attenuated but remained after adjustment for covariates. However, the relationship between EPA and total fat and PUFA from fish-shellfish disappeared in men. Further, there was a dose–response relationship between the three-level weekly fish intake and total *n*-3 fatty acids in plasma PL in both men and women (ANOVA *P* < 0·001; data not shown).

**Table 5.** Crude Spearman rank correlations (*r*) between specific plasma phospholipid fatty acids and dairy products and meat and in all study participants (*n* 279) and by sex

		Fish and shellfish† (g/d)			Total fat from fish and shellfish† (g/d)			PUFA from fish and shellfish† (g/d)			Weekly fish intake‡		
Fatty acid		All	Women	Men	All	Women	Men	All	Women	Men	All	Women	Men
Total *n-*3		0·35***	0·36***	0·35***	0·34***	0·39***	0·29***	0·33***	0·38***	0·29***	0·45***	0·50***	0·38***
20 : 5*n*-3		0·31***	0·33***	0·29***	0·26***	0·34***	0·17*	0·25***	0·32***	0·17*	0·41***	0·47***	0·30***
22 : 6*n*-3		0·36***	0·36***	0·34***	0·38***	0·42***	0·34***	0·37***	0·42***	0·33***	0·44***	0·47***	0·40***

*P ≤ 0·05, ***P < 0·001.

†Residual adjusted.

‡From questionnaire.

The ranking of the fish and shellfish intake in the questionnaire was also fair in comparison with the intake of fish-shellfish from the diet record (κ_w_= 0·30; *P* < 0·001).

Meat and sausage intake was negatively correlated with plasma PL 14 : 0 (*r* –0·20; *P* = 0·02) in men and 15 : 0 (*r* –0·18; *P* = 0·03) in women, but not with PL 17 : 0 in either women or men. These correlations were unaffected by adjustments. Intake of spreads did not correlate with any of the plasma PL fatty acids, except for a marginal correlation with 15 : 0 (*r* 0·11; *P* = 0·06) (data not shown).

In sensitivity analyses, when under-reporters and supplement users were excluded, respectively, the results were only marginally different (data not shown).

## Discussion

The present study indicates that the web-based 4-d food record, the Riksmaten method, used in the latest Swedish National Dietary Study in adults, appears valid for the purpose of collecting information on dietary fat. Moderate associations between the consumption of longer-chained *n*-3 fatty acids as well as fish-shellfish intake and PL levels are reported. Further, the intake of fish and shellfish reported in the questionnaire correlated with the intake of fish and shellfish from the diet record as well as with plasma EPA and DHA. We also found a dose–response relationship between fish consumption and levels of *n*-3 fatty acids in the blood, consistent with previous studies^(^^27^^,^^28^^)^. The present study also supports that myristic acid (14 : 0) is a valid marker for dairy food intake, but surprisingly we did not find a relationship between PL 14 : 0 and dairy fat. This may be because myristic acid only makes up a small proportion of dairy fat^(^^29^^)^ and that 14 : 0 is present in other dietary sources such as coconut oil. We did, however, find a significant positive correlation between PL 14 : 0 and dietary 14 : 0. Pentadecaenoic acid (15 : 0) was correlated with both intake of dairy products and amount of fat from dairy products while heptadecaenoic acid (17 : 0) was related to the amount of fat from dairy products, mainly in women.

The proportions of plasma PL fatty acids reported in this national dietary study were in the same range as reported in other Swedish studies and analysed in the same laboratory^(^^30^^–^^32^^)^. However, the proportion of plasma DHA in the present study was lower compared with that reported in previous Swedish studies^(^^30^^–^^32^^)^. Participants who had not eaten fish during the registration period had lower proportions of plasma EPA and DHA, which is consistent with previous observations^(^^27^^)^, and possibly explains the detected lower proportions in the study population. The proportions of EPA and DHA were positively related to age and this is in agreement with a higher consumption of fish-shellfish at higher ages^(^^7^^)^. Those who consumed fish-oil and *n*-3 supplements had higher levels of EPA and DHA, but reported likewise a higher intake of fish-shellfish. Less than 20 % of the study population used supplements and exclusion of these individuals from the analysis had only marginal effects on the results.

There was no correlation between dietary and plasma PL LA when all study participants were examined, but there was a marginal correlation in women (*r* 0·15; *P* = 0·06). This is in contrast to previous studies^(^^25^^,^^26^^)^, but not in all^(^^33^^)^. It is possible that the diet record did not adequately measure LA intake, or that the intake range was too narrow to detect a correlation. A preferential misreporting of foods high in LA is possible. LA was the largest individual PUFA in the diet and is a precursor for longer and more unsaturated *n*-6 fatty acids like arachidonic acid (20 : 4*n*-6), a precursor for eicosanoids^(^^15^^)^. This endogenous conversion as well as oxidation of LA may have affected the correlation between diet and biomarker. We could not retrieve information about the amount and type of cooking fat used in dishes and for baking from the diet record, due to technical reasons. It is possible that this may partly explain the lack of correlation between PL and dietary LA. Also, the use of standard fat in recipes in the diet record may have influenced the result. These reasons may explain the lack of correlation between dietary α-linolenic acid (18 : 3*n*-3) and plasma levels in the present study as well.

That tissue SFA is weakly correlated with diet is partly related to the rapid endogenous conversion to MUFA from SFA by desaturases^(^^21^^)^. Further, MUFA was negatively correlated with dietary intake partly because of the endogenous production of MUFA. Biomarker MUFA correlated with SFA and this has been reported previously^(^^26^^)^. This relates to the fact that the intake of oleic acid in Sweden, as well as in for example the USA, is mainly derived from dairy products and meat which also are main sources of SFA.

In the present study, plasma PL 15 : 0 and 17 : 0 were correlated with the intake of dairy products and partly to dairy fat as previously suggested^(^^24^^,^^34^,^35^^)^, although the correlations differed between the sexes. Different species of fish contain 15 : 0 and 17 : 0 of varying content^(^^36^^)^ and a previous study (European Prospective Investigation into Cancer and Nutrition; EPIC) reported positive associations between tissue 15 : 0 and 17 : 0 and fish intake on an ecological level^(^^37^^)^. However, we found no correlation between milk fat biomarkers and fish intake in the present cross-sectional study.

The intake of meat and sausages was not correlated with tissue 15 : 0 and 17 : 0, probably because the intake of pork and pork-containing sausages exceeds the intake of ruminant meat in the survey.

The magnitude of the correlations between individual fatty acids in PL and diet was in the present study as what can be expected with only one diet measurement and in the same range as previously reported^(^^25^^,^^26^^,^^38^^)^. Correlations between fatty acids measured in plasma PL and diet differed in men and women, despite similar proportions in plasma. It is possible that these results are due to chance or it is possible that fatty acids from different dietary sources affect metabolism differently. Further, it is possible that accuracy of reporting is different among men and women, which was partly confirmed in the interaction analysis. It is further known that fat metabolism is different in men and women^(^^39^^)^. Non-dietary factors said to affect plasma fatty acid composition^(^^21^^)^, such as adiposity and age, differed between the sexes and may have influenced the results, but this was accounted for in the analyses.

The strengths of the present study include the random population sample that included women and men from all parts of Sweden, the careful building of the food list in the web-application and that data was quality controlled before analysis. Further, the web-based dietary record was self-assisted, which minimised external coding errors. This could be a limitation since self-reporting could be considered as if there were 279 individual reporters/coders. However, this introduces a random error whereas using interviewers for coding could introduce a systematic error. On the other hand, interviewers might be able to retrieve more detailed information on foods compared with self-reporting. Another limitation is that the participation rate was low; this could have introduced bias. Participants had a higher degree of educational attainment than those who did not participate and few subjects with immigrant background participated^(^^7^^)^. This limitation relates, however, more to the dietary survey rather than to the present relative validation. Under-reporting may influence the results in any diet study, but when under-reporters were excluded from our analyses the interpretation of the results remained unchanged. Another limitation is that we did not measure fatty acids in any other fraction, for example the cholesteryl ester fraction. It is possible that our 4-d diet record did not reflect the fat intake representing the same period as the fatty acids in the PL fraction.

Dietary data (food consumption data) collected in a national dietary survey are used for several purposes: to monitor nutrient and food intakes in the population as well as to carry out food-based risk–benefit assessments and policy making within the European Union. It is therefore important that the data collected meet the requirements set out both on a national level as well as by the European Food Safety Agency^(^^40^^)^ and are as accurate as possible. In conclusion, the Riksmaten method appears valid for the purpose of collecting data on dietary fat composition derived from several core food groups, at least in a healthy adult population.
